# Patient Perspectives on Impact of Weight and Weight Stigma on Breast and Cervical Cancer Treatment: A Qualitative Study

**DOI:** 10.1002/cam4.70823

**Published:** 2025-03-20

**Authors:** Jamie L. Sorensen, Michele M. West, Kathleen M. Robinson, Mary E. Charlton, Ingrid M. Lizarraga, Sarah H. Nash

**Affiliations:** ^1^ Department of Epidemiology, College of Public Health University of Iowa Iowa City Iowa USA; ^2^ State Health Registry of Iowa, College of Public Health University of Iowa Iowa City Iowa USA; ^3^ Holden Comprehensive Cancer Center University of Iowa Iowa City Iowa USA; ^4^ Division of Endocrinology and Metabolism University of Iowa Hospitals and Clinics Iowa City Iowa USA; ^5^ Department of Surgical Oncology, College of Medicine University of Iowa Iowa City Iowa USA

**Keywords:** body mass index, cancer treatment, weight stigma

## Abstract

**Background:**

Higher weight individuals report experiencing weight‐based stigma in the healthcare setting; within the cancer continuum, the most robust evidence exists for cancer screening. More research is needed to understand whether and how higher weight patients experience weight stigma during cancer treatment.

**Methods:**

We conducted semi‐structured interviews with 15 breast and 15 cervical cancer survivors diagnosed 2017–2019 in Iowa who had a pre‐diagnosis body mass index of 30+ kg/m^2^ calculated from their driver's license height and weight. Interviews focused on whether individuals perceived being treated differently because of their weight in daily life, in healthcare, or during cancer treatment. Data were coded using a combination of inductive and deductive approaches, and analyzed using a multi‐phase thematic analysis.

**Results:**

Almost all interviewees reported positive experiences during cancer treatment; several described their weight as never being an issue. Some identified weight stigma during cancer diagnosis or treatment that resulted in delayed diagnoses or changes in treatment. Many interviewees described situations where their weight was discussed negatively during cancer treatment, but most did not identify these as stigmatizing because their providers were only “concerned about [their] health.” Additional themes developed included experiencing environmental stigma, the discussion of cancer recurrence by providers only as it related to weight, and misconceptions of the causes and consequences of obesity.

**Conclusions:**

While several participants did not feel that their weight impacted cancer treatment, some reported experiences of weight stigma pre‐diagnosis and during treatment. When individuals noted their weight was discussed during treatment, internalized bias may have impacted whether they considered these discussions stigmatizing.

**Abbreviation**
BMI: body mass index

## 1 Introduction

Women in larger bodies may be at higher risk of developing certain cancers, including breast and cervical cancers [1, 2] and may experience lower cancer survival [3, 4, 5]. Possible mechanisms for survival differences may relate to reduced access and use of cancer screening, resulting in later stage diagnoses; [6, 7] effects of obesity on estrogen metabolism and efficacy of anti‐estrogen medication; [8, 9] more biologically aggressive tumors [10, 11]; and differential chemotherapy dosing due to concerns of increased toxicities [12, 13, 14]. Further, obesity may be associated with increased risk of other comorbidities that may impact a patient's ability to receive and complete all modalities of cancer treatment [15], although multimorbidity associated with cancer and obesity is less well described. The prevalence of obesity among women with cancer is ~30% and increasing over time [16]. Given that very few weight loss interventions have proven effective at helping people lose weight *and maintain that weight loss* for more than 5 years [17], the need to understand contributors to observed survival disparities is critical in order to achieve optimal health outcomes for those in larger bodies, independent of weight loss efforts.

Experiences of weight stigma may also influence access to and utilization of healthcare by those in larger bodies [18], in turn affecting cancer outcomes. This includes healthcare avoidance, which is when patient disengagement causes an individual to delay or avoid seeking healthcare [19, 20, 21]. Weight stigma is the social devaluation, denigration, and differential treatment of individuals based on their body weight [22, 23]. Healthcare providers are a primary source of weight stigma; evidence of this is consistent across multiple study populations and countries [18, 21, 24, 25].

The strongest evidence for the impact of weight and weight stigma on care across the cancer continuum (i.e., the time from screening, to diagnosis, to treatment, to cancer‐related outcome) is in cancer screening. For example, women in larger bodies are less likely to receive Pap screening for cervical cancer than those in smaller bodies [26] and are less likely to be diagnosed with cervical precancers and more likely to be diagnosed with cervical malignancies [2]. Further, while obesity is a risk factor for postmenopausal breast cancer [27], women with obesity are less likely to receive mammography [28] or clinical breast exams [29] and are more likely to be diagnosed with breast cancer at later stages [30]. Women in larger bodies delay cancer screening for reasons including disrespectful treatment, embarrassment at being weighed, medical equipment that is too small, and negative attitudes of providers [7]. Lack of access to cancer screening and/or engaging in healthcare avoidance may delay treatment initiation for cancer among individuals with overweight and/or obesity [31]. Such delays may have serious implications that could contribute to observed cancer survival disparities [5] cancer survival is lower at later stages at diagnosis for almost all cancer sites, and treatment delay is associated with a 6%–23% increased mortality risk [32, 33].

Although up to two‐thirds of higher weight individuals report having experienced weight stigma in the clinical setting, [21] few studies have described patient experiences in cancer treatment, [24] and the prevalence of weight stigma experienced by these patients during cancer treatment is unknown. A small number of qualitative studies indicate that stigmatizing experiences do occur among cancer patients and survivors with obesity, including instances of being offered less invasive treatment options (e.g., lumpectomy versus mastectomy), mistreatment, and lack of available equipment to accommodate larger body sizes [34, 35, 36]. Qualitative research has also indicated the potential for weight bias internalization (i.e., internalization of stigmatizing beliefs about obesity among those with obesity) among cancer patients with obesity, [36] which may lead to healthcare avoidance and/or undertreatment [18, 20, 21]. To add to this sparse literature base, we sought to understand both the impact of weight and experiences of weight stigma among breast and cervical cancer survivors living in the Midwestern U.S. throughout their cancer journey. We were interested in where along the cancer continuum these experiences occurred, and specifically whether/how they impacted cancer treatment.

## 2 Methods

### 2.1 Ethics Statement

This study was approved by the University of Iowa Institutional Review Board (IRB #202205265). Participants provided verbal informed consent prior to interviewing. A waiver of documentation of consent was approved by the University of Iowa Institutional Review Board.

### 2.2 Study Population

This study recruited breast and cervical cancer patients identified using the Iowa Cancer Registry. The Iowa Cancer Registry has been part of the National Cancer Institute's (NCI) Surveillance, Epidemiology, and End Results (SEER) Program since its inception in 1973. Potential participants (*N* = 129 [*N* = 57 breast cases, *N* = 72 cervix cases]) were eligible for recruitment if they were at least 18 years of age, diagnosed with histologically confirmed breast or cervical cancer (ICD‐O‐3 codes C500‐C509 for breast, and C530‐C539 for cervix) between 2017 and 2019 (i.e., most recent complete cases available through the registry), received treatment for their cancer, were alive and eligible for contact, and had a body mass index (BMI) above 30.0 kg/m^2^. The time‐window was restricted to reduce recall bias, and increase the likelihood that participants would remember information about their treatment experience. Information on BMI was obtained from Iowa Cancer Registry data linked with driver's license data to obtain height and weight, calculated as weight (kg)/height (m)^2^. Weight at the time of diagnosis was confirmed with the participant at the beginning of each interview. Participants were only eligible if their driver's license was valid within the 3 years prior to their cancer diagnosis.

### 2.3 Study Recruitment

Study recruitment began in May of 2022 and continued through July of 2022. Potential participants (*N* = 129) were contacted in batches until the target number of participants had been reached; participants were selected to equally represent breast and cervical cancer patients (breast, *n* = 15, cervix, *n* = 15). The number of participants was chosen a priori and was based on the concept of information power, [37] informed also by the available time and resources to complete interviews. Those who were eligible for study participation received a mailed study invitation letter, followed 2 weeks later by a phone call from a member of the study team to provide more information about the study, obtain informed consent, and conduct or schedule the interview if amenable. During this phone call, participants were re‐assessed for eligibility using the criteria listed above. Participants who did not respond to the initial study invitation letter were contacted up to six times via telephone to discuss their potential study participation.

### 2.4 Interview Guide Development

The interview guide was developed iteratively with input from all members of the research team. Briefly, the first draft of the interview guide was developed by JS and SHN, reviewed by study team members IL, MEC, and KR, and iteratively revised until consensus was reached. Each interview question was selected to directly address one of the following pre‐defined research questions: (1) How have breast and cervical cancer survivors in larger bodies experienced weight stigma in their daily lives? (2) How have breast and cervical cancer survivors in larger bodies experienced weight stigma in the healthcare setting? Where did those experiences occur (e.g., primary care, specialty care, etc.), and how did they respond to those experiences? (3) How have breast and cervical cancer survivors in larger bodies had body‐positive experiences or positive experiences within the healthcare setting? Where did those experiences occur (e.g., primary care, specialty care, etc.), and how did they respond? (4) What barriers to receiving a cancer diagnosis and/or treatment did the individual experience? (How) did weight and/or weight stigma impact those barriers? (5) (How) did the individual's weight affect their cancer diagnosis or treatment, or their interactions with healthcare providers during the diagnosis and treatment process? While not directly pertinent to our research questions, we also asked participants to describe instances of weight stigma in their daily lives to determine whether they understood the concept and were able to identify personal experiences of weight stigma. Interview questions and detailed rationale are provided in Table S1.

### 2.5 Interview Procedures

A paper copy of information about the study was provided to each participant with the initial contact letter sent ahead of the interview so that participants were able to review, discuss with friends and family, and bring any potential questions to consent and interview. All interviews were conducted via phone call. Before beginning each interview, if participants requested, the interviewer would read the study information sheet, including consent information. All participants gave permission to have their interview recorded. JS, a graduate research assistant trained and experienced in qualitative research methods, [38] conducted all interviews, asking clarifying questions, documenting salient findings, and probing for additional information as needed. She had no prior relationship with the participants. Interviews lasted approximately 15–30 min; the mean interview time was 20.8 min (minimum: 6 min, maximum: 70 min). Interviews were recorded in duplicate using handheld audio recording devices. Recordings were then transcribed using Rev., [39] a secure online transcription service; all identifying data were removed from transcripts by the researchers before analysis. Interviewees were compensated $30 for their time and participation.

### 2.6 Data Analysis

Qualitative data were analyzed using a multi‐phase thematic analysis [40]. All qualitative analyses were conducted using Dedoose version 9.0.86 (SocioCultural Research Consultants LLC, Manhattan Beach CA). Two study team members (JS, SN) developed a list of a priori codes, informed by previous literature on weight stigma in the healthcare setting [18, 19, 20, 21, 22, 23, 24, 25]. Additional emergent codes that arose from reviewing the data were defined based on an initial review of five transcripts (JS, SN), and the codebook was finalized. All transcripts were then (re)coded using an iterative process: initial coding was completed by JS; SN then reviewed all transcripts and codes, and flagged codes for discussion where there was any disagreement; JS and SN then discussed any discrepancies until consensus was reached. Themes were identified through an iterative process of data visualization and reduction (JS, SN). First, we condensed overlapping codes and removed unused codes. We then reviewed all codes to determine areas of co‐occurrence and possible reduction into themes. Next, we identified frequently used codes and codes with novel insight to determine if these codes represented standalone themes, and summarized and categorized the quotations within each code and theme. Breast and cervical cancer survivor interviews were analyzed together. Themes were also compared between cancer sites to determine whether any major differences existed. Themes were identified by JS and SN and presented to the rest of the research team for discussion until agreement was reached.

### 2.7 Positionality

This research was conducted by individuals who hold identities and experiences that impact the way they engage with research and interpret research findings. The research team actively acknowledged and considered their relative position to the participants of this study throughout its conduct. Relevant experiences include one team member with obesity who has experienced weight stigma in the clinical setting, academicians (all team members), and clinicians (two individuals; one endocrinologist, one surgical oncologist); none have personally experienced a cancer diagnosis.

## 3 Results

### 3.1 Participant Characteristics

Of 129 eligible participants, 30 consented to participate and completed an interview. A summary of participant characteristics is provided in Table 1. All study participants identified as female, and the average age of diagnosis was 54 years (min = 32, max = 77 years). The average self‐reported BMI at diagnosis for study participants was 42.7 kg/m^2^ (min = 30.0, max = 76.38); for breast cancer patients, this was 42.97 kg/m^2^, and for cervical cancer patients, it was 40.84 kg/m^2^. The average time since diagnosis for all participants was 4.4 years (min = 2, max = 6 years); for breast cancer patients, this was 4.73 years, and for cervical cancer patients, it was 4.33 years. A small number of individuals reported co‐occurring cancer diagnoses (6.7%); however, interview questions and answers focused exclusively on the cancer site of interest for this study (i.e., breast and cervix).

**TABLE 1:** Demographic and clinical characteristics, Iowa breast and cervical cancer survivors in larger bodies, 2022, *n* = 30.

Variable	*n*	Mean ± SD (Range)
Age (years)	30	53.6 ± 11.45 (32–77)
BMI	29	42.7 ± 9.95 (30–76.4)
Weight^a^	29	248.8 ± 62.8 (170–445)
Height^b^	30	63.8 ± 2.17 (60–68)
Time since diagnosis^c^	30	4.4 ± 0.99 (2–6)

	*n*	%
Cancer type		
Breast	15	50.0
Cervix	15	50.0
Multiple diagnoses		
Yes	2	6.7
No/Unknown or undisclosed	28	93.3

^a^ Weight measured in pounds, self‐reported during the interview.

^b^ Height measured in inches, self‐reported during the interview.

^c^ Time since diagnosis measured in years, self‐reported during interview.

### 3.2 Interview Findings

All interviewees (*n* = 30) reported having positive experiences during the process of cancer treatment. While some individuals reported experiences of stigma prior to or during treatment, several described their weight as never being an issue during the receipt of care (*n* = 21).

The main themes identified from our interviews included (1) the impact of weight on cancer treatment and treatment decision‐making, (2) instances of weight stigma in daily life, healthcare, and during cancer care, (3) patient responses to stigma, (4) clinical discussion of weight in the context of cancer recurrence, (5) participant attitudes towards their body size, and (6) misconceptions around causes and consequences of obesity. We also identified experiences that were unique to either breast or cervical cancer survivors. Theme definitions, examples of representative codes, and quotations for each of the identified themes are provided in Table 2.

**TABLE 2:** Identified themes and representative quotations, Iowan breast and cervical cancer survivors in larger bodies, 2022, *n* = 30.

Theme (code)	Selected quotes
Impact of weight (stigma) on treatment, and treatment decision making *Definition: Whether and how it was perceived that the individual's weight affected their ability to receive a cancer diagnosis or treatment*.	
No impact of weight on diagnosis or treatment	“No. Even with all the scans and the tubes and stuff, it was never an issue.” “I don't feel like my weight ever factored into any care I've ever had.”
Weight impacted diagnosis and/or treatment	“I pretty much, yeah, just the MRI. I mean, if I had to go back to the MRI, one time they almost sent me home because they were thinking they weren't going to be able to get me into it. I think the MRI was the only thing that's almost stopped me from getting something done because of my weight.” “My surgeon said, “I don't advise that, I like to make sure that it's the least invasive surgery possible and I just want to do a lumpectomy.” I said, “Well, I would like to have a mastectomy and then allow for reconstruction.” And she said, “I won't…I am recommending a lumpectomy.” I said, “Why don't you want me to do the other?” She said, “Well, you have diabetes and that increases the risk of the healing process.” And so the diabetes is because of my weight. …And then after the surgery, the pathologist said that there was evidence of cancer cell too close to the outer limit of where she took the tumor, whatever it was, the cancer. And so they told my surgeon she had to go back in and do it again. And when I went back in to reschedule, to do the second surgery, I said, “If you'd let me do the mastectomy we wouldn't be here today.”
Treatment decisions and/or outcomes (identified by participant) that were deemed weight‐related by a provider, but were not actually weight‐related	“It [weight] made it [surgery] a little more difficult because most of my weight is in my belly and my back side and my upper legs. It didn't help any being overweight. …But I've got scars from my car accident and stuff. So there's a lot of extra scar tissue inside [my abdomen].” “I have a bigger stomach, but soon after my surgery, my incision broke open. … And my doctor explained to me that my weight, we had several talks about weight and affecting the surgery, but he was very, very nice about it. …Well, that, they found out later I was allergic to these sutures, and so my body would just spit them out.”
Instances of weight stigma in daily life, or healthcare *Definition: Any experience, actual or perceived, that resulted in feelings of mistreatment, neglect, unfairness, inadequacy, or disgrace as a result of the individual's weight. Occurring outside of the cancer continuum*.	
Everyday life	“I don't look the part. I applied for a bartender job and I was denied and later found out that if I would've had a body that I could have wore a more revealing shirt, I would've gotten the job.” “Oh, yeah. I used to be a bus driver for the school. And a lot of kids would say, “There's that fat lady coming.” Some kids would come out and say, “Well, where's the bitch, that fat bitch?” And I had to learn to ignore them.” “I went to Planet Fitness to see about getting a trainer to help me with my weight. So, they was helping me fill out papers and stuff like that, but what turned me off from them is when I was going to the washroom, and I heard the lady say, “Her big ass going to need a lot of training.”
Primary healthcare setting	“Yep. I was told by one of my main physicians that I was seeing at the time I found out I was pregnant. She told me that if I didn't lose weight, I was going to kill myself and my child.” “Well, when I've had to see some other physicians, you can definitely tell that they have, I don't know if you'd want to say an attitude or a judgment to someone who is overweight, in the sense that they assume if you're overweight that all you do is sit and eat McDonald's and junk, which isn't my case at all. But they kind of put off that message to you. That you don't know what you should be eating or all you do is sit all day long because you're overweight.” “I was diagnosed with PCOS and the first thing that the doctor had talked about when we were going through infertility treatments was that I needed to lose weight or I would never become pregnant.” “She said, ‘If you lose weight, you probably don't have to be on this type two diabetes medication. We can probably get you … I think it's really a direct reflection of your weight and maybe even your blood pressure.’” “I mean, nothing. It wasn't referred to except that “you really should probably lose some weight.”
Instances of weight stigma during cancer diagnosis and treatment *Definition: Any experience, actual or perceived, that resulted in feelings of mistreatment, neglect, unfairness, inadequacy, or disgrace as a result of the individual's weight. Occurring during the cancer continuum*.	
Environmental weight stigma	“I would say the only thing that I personally had issues with was, I had to have several MRIs done, and I always asked to have the fat lady machine. Again, I'm my own worst critic. But I always ask for that because if I ever had to go have my MRI done in the normal skinny people machine, I was just crazy claustrophobic so I had to have that bigger machine.” “Some of them would use two gowns. They put one on my back and then I'd wear the other one backwards so that I was covered. But when you first get into the dressing room and your only choice for a gown is one that won't fit and you have to, I mean, I didn't know what to do. So I had to sit there and try and clench it shut and wait for a nurse to come back and then when she realized, oh yeah, you don't fit, here, let's put one on backwards just to cover you up.”
Interpersonal/provider weight stigma	“Well, this was even a doctor. The doctor that was going to do the surgery. They just said that I carry a lot of weight down there [abdomen], but I probably should have surgery just to look better.” “And you could just tell sometimes, the radiologist there, not the radiologist, but the workers that do it every day, I had one make a comment about how big my stomach was.” “You're sitting there with no top on, and then he [plastic surgeon] is like, “Oh yeah, you are big. He goes, “There won't be any problem [with a reconstruction].”
Unintentional/implicit weight stigma	“They want you to lose weight to help your health, but they don't offer much advice on how to do that correctly.” “No, just the obvious like when you have your appointments, they always talk about the healthy weight type of thing.”
Intentional/explicit weight stigma	“And then when it comes time to move you over to the surgery table, they say they're going to inflate it and it just helps to move you. So then when I get in there it was actually the anesthesiologist. Because I'm still awake when they take you in. And he just said, “Well, can't she just move over by herself?” Just kind of crass like that. No bedside manner. And the nurse was amazing and she's like, “Oh no, I have this. This is what we do.”
*Patient responses to weight stigma* *Definition: Any action or emotion from a participant that occurred as a result of a stigmatizing experience. May or may not have occurred during the cancer continuum*.	
Attitudes/emotions	“I've always been self‐conscious, so it just kind of brought that out in me.” “I guess that's just something that always kind of sticks with you though.” “[It makes them feel] less than others. That there's a fault with me that I can't… Oh, there's all kinds of… You feel like a lesser quality human being, I guess.”
Action steps	“I've since switched doctors so I'm not with that one anymore.” “I told her some words I can't say over the phone.” “I come back at them and said, ‘hey this my life, not yours” “I wear clothes that are not necessarily the ones I like, but that fit my proportion to my body. I go to Goodwill, and Salvation Army, and get clothes that I don't wear something tucked in because then that exceeds, that shows off my stomach, and I'm sure I have no waistline.”
*Clinical discussion of weight in the context of cancer recurrence* *Definition: Any mention of weight that occurred in a medical setting. Includes positive, neutral, and negative discussions, and may or may not constitute a stigmatizing experience*.	
Discussion of weight related to cancer recurrence	“My doctor said, ‘If you gain this weight back, it's a 50/50 chance your cancer could come back.’ So I'm really like scared of that, so I'm really trying my best to lose some weight.” “The only real time that it [weight] was talked about, to be honest, was when she said … and she said it at a couple of different visits, but that weight can be a contributing factor in cancer, that they've been finding that.”
Personal attitudes towards their body size Definition, personal attitudes: A settled way of thinking about something. *Definition, internalized bias: Self‐stigmatizing thoughts or beliefs about one's image/appearance or weight; an individual's understanding or interpretation of their weight, image, or appearance that reflects weight stigma*.	
Weight loss a “silver lining” of cancer	“I had hoped that with the treatments that I would end up losing weight. I dealt with so many weight issues that it was a feeling of, with everything else that was going on, if my weight were to drop because of that, then that would be a blessing in a terrible situation.” “It was kind of depressing when I found out that I only lost maybe about seven pounds because of taking them [breasts] off, I was hoping to lose more.”
Internalized bias	“Obviously, I know I'm overweight. I have a mirror.” “I'm like, yeah, I know I got to get a grip.” “Well, just seeing pictures of myself. I lost my hair, lost my eyebrows and stuff like that and just sometimes being in pictures and seeing myself, I was really just disgusted with myself. And of course those pictures come back around, around anniversaries and things like that. Social media and stuff like that and you see that stuff and you're just like, oh my gosh. Yeah. It really just bothered me, like I said, that I let it [weight] get that far.”
Attitudes towards fatness	“I know that I did this… I know the weight thing I know I did it to myself, I know that it wasn't cancer.” “And you're going to sit by someone, and you wonder if they go, “Oh, great. I got the fat chick.” I mean, I'll think that sometimes of even bigger people than me.”
*Misconceptions of causes and consequences of obesity* *Definition: A view or opinion on obesity that is incorrect or misguided because of its basis on faulty thinking or understanding; an inaccurate idea or conception of obesity*.	
Participant‐held misconceptions	“So I know for sure it was nothing to do with cancer, I know for sure that it's something I did to myself.”
	“I know that I did this… I know the weight thing I know I did it to myself.”
Provider‐held misconceptions	Some doctors can be like, “Okay, you need to do this, or you're going to die, basically.” “I got a new general practitioner. And she was a little upset about my records, it didn't look like I had been being very careful about my diabetes. And she said, “I'm disappointed that you have not been in better control than you've been. And that will be our goal to get you in control and get your A1C and everything as good as possible.” And so part of that was making sure I had medication that would… Well, she always was asking about food control and weight and activity. She was very concerned, but in a good way, about saying, “If you lost more weight, your diabetes would surely be better.”
Justification of discussion of weight from providers as “deserved”	“And I didn't say another word because obviously I was fat. I didn't feel sad, I didn't feel angry, I just felt, “Oh, okay.” I accepted the reason for this new medication.” “I guess in a way, I mean, I understand why it's being brought up. I mean, I completely understand why it's being brought up and everything and I don't feel ashamed as they're bringing it up or anything like that, but it's sometimes it's just hard to hear it from someone else.”

### 3.3 Impact of Weight (Stigma) on Cancer Treatment, Treatment Decision Making

Most individuals reported that they did not believe their weight had an impact on cancer treatment: “…basically, they were more focused on my treatments, treatment plan, my side effects, things like that. Yeah, like I said, I don't recall them ever really mentioning my weight at all during treatments.” Many participants reported that when their weight was mentioned, it was addressed in a “matter of fact” manner that participants did not attribute to differential treatment.

Some participants did report an impact of their weight and/or body size on cancer treatment; some of these experiences were perceived as stigmatizing, but most were not. For example, one woman (cervical cancer) reported her provider switched from a traditional hysterectomy to a laparoscopic procedure in order to reduce the risk of weight‐related complications while under general anesthesia. Further examples are given in Table 2.

### 3.4 Experiences of Weight Stigma in Daily Life, and Healthcare

Nearly all participants were able to give an example of experienced weight stigma outside their cancer care experience. One participant said, “There have been a few instances in my life when I felt like people were looking at me and were kind of… I don't want to say disgusted, but something like that.”

Many participants reported experiencing weight stigma in the healthcare setting; these manifested as both explicit and implicit weight stigma. While some stigmatizing experiences were obvious, (“I was told by one of my main physicians that I was seeing at the time I found out I was pregnant. She told me that if I didn't lose weight, I was going to kill myself and my child”), others were more subtle (“They want you to lose weight to help your health, but they don't offer much advice on how to do that correctly”). These instances of weight stigma, perhaps unintentional in nature, often still left a negative impact on participants. Indeed, some participants who reported experiencing weight stigma in the clinical setting seemed to feel unsure about what had transpired, and while they felt uneasy about the situation, often dismissed their unease as providers were “only concerned about [their] health”; for example, “[the provider] said, ‘Well, you're really only hurting yourself.’ And I said, ‘I understand that.”

### 3.5 Experiences of Weight Stigma During Cancer Diagnosis and Treatment

Some participants identified weight stigma occurring at points throughout the cancer continuum. For example, one woman noted that her provider talked about her weight before delivering her cancer diagnosis: “I just found it horrific… that the doctor was going to tell me for sure that I had cancer, and she already knew that, but she didn't tell me first. She was commenting on the fact that I had some irritation and a possible yeast infection in the crease under my belly.” When weight stigma was reported during cancer treatment, it was both environmental (i.e., inadequately sized medical equipment) and interpersonal (i.e., stigmatizing behavior from a medical provider) (Table 2).

### 3.6 Patient Responses to Stigma

Individual responses to encountering a stigmatizing experience in health and cancer care were varied and could be classified either as an emotional response or an action taken. Most participants reported emotional responses; for example, heightened awareness and a sense of hyper‐vigilance around providers who discussed weight and feeling the need to defend themselves. The most commonly reported actions taken were doctor shopping, calling out the provider, or modifying their habits to reduce the chances of further stigmatizing experiences (Table 2). Lastly, as a result of stigmatizing experiences, some women reported completely avoiding the healthcare system and that they put off receiving healthcare until very dire circumstances, typically to avoid weight‐related scrutiny.

### 3.7 Clinical Discussion of Weight in the Context of Cancer Recurrence

Participants reported that when weight was discussed in the cancer treatment setting, it often was included in discussions of cancer recurrence. Some participants indicated that their cancer providers would only share statistics of cancer recurrence as they related to their weight or weight gain (Table 2). Participants reported that general recurrence risk statistics were not shared (i.e., how likely the cancer is to come back regardless of weight).

### 3.8 Participant Attitudes Towards Their Body Size

Some participants expressed personal attitudes or beliefs regarding obesity, and specifically their own body size. Instances where these attitudes were presented often followed the mention of a stigmatizing experience that occurred, and frequently indicated sentiments of internalized weight bias. Further, some women discussed the concept of weight loss as a silver lining for cancer, indicating that their weight loss would be a “good” thing to come out of their cancer battle. Examples are presented in Table 2.

### 3.9 Misconceptions Around Causes and Consequences of Obesity

Misconceptions around the causes and consequences of obesity were central to most interview responses (Table 2). Misconceptions were reported for both providers and participants themselves, and were mostly comprised of participants reporting that either they or their physician understood obesity as a controllable, individual choice that could simply be reversed if the individual tried hard enough.

### 3.10 Differences in Weight Stigmatizing Experiences by Cancer Site

While we observed similar themes across both breast and cervical cancer survivors, there were some experiences specific to breast or cervical cancer, respectively.

Specific to cervical cancer survivors were discussions to weight stigma as it related to fertility. One participant reported seeking a referral for fertility treatment for many years and being repeatedly dismissed and told she just needed to lose weight; this participant was eventually diagnosed with polycystic ovary syndrome (PCOS). This same participant went on to receive a cervical cancer diagnosis years later, again after being dismissed as only experiencing health problems due to her excess adiposity; this participant reported only being offered care after substantial weight loss (i.e., the provider recommended weight loss before further exploring her symptoms). Further, cervical cancer survivors reported stigmatizing experiences that were not weight‐based, for example, derogatory comments about cervical cancer and sexual health, which were not reported by breast cancer survivors.

Unique to breast cancer survivors were reports of stigma with regards to surgical reconstruction. For example, one woman was recommended a breast reduction in addition to a complete reconstruction without indicating her desire for this treatment. Another woman, when consulting with a plastic surgeon, was told that she had “more than enough excess skin and fat” for a reconstruction to be successful, before indicating whether she would like to seek a reconstruction following her mastectomy.

## 4 Discussion

In this study, we sought to understand experiences of weight stigma among breast and cervical cancer survivors in larger bodies, where along the cancer continuum these experiences occurred, and specifically whether/how they impacted cancer treatment. While most women in our sample reported that they perceived their weight not to have impacted their cancer treatment, some did report stigmatizing experiences; these could be classified into environmental (e.g., MRI machines that were not big enough) or interpersonal (e.g., a provider who discussed a patient's weight before sharing their cancer diagnosis) stigma. Reported responses to stigmatizing experiences were both emotional (e.g., heightened awareness of their weight), and action‐oriented (e.g., doctor shopping, healthcare avoidance, or pushing back against providers). When weight was discussed during treatment, it was often reported in the context of cancer recurrence i.e., that losing weight would result in lower risk of recurrence; few participants identified these discussions as stigmatizing, noting that providers were only concerned about their health. Finally, nearly all themes identified from our analysis related in some way to misconceptions around the causes and consequences of obesity by either patient or provider (or both); specifically, that obesity solely occurs as a result of an individual's personal health behaviors and actions and is a controllable, individual choice that can be simply reversed if the individual tries hard enough, without regard for the myriad of complex causes of obesity [41, 42]. From the participant perspective, this misconception was often twinned with sentiments of self‐blame and internalized stigma. Together, our findings indicate that while many women with obesity may not identify any impacts of their weight on breast or cervical cancer treatment, for those who did report stigmatizing experiences, the impact was likely profound.

It is important to acknowledge that the discussion of weight in the clinical setting may be a necessary component of care; however, it is crucial to discuss weight with patients in a sensitive and compassionate way. Repercussions of discussing weight in a manner perceived as stigmatizing to the patient may result in healthcare avoidance, doctor shopping, and/or a lack of timely care for individuals with obesity [18, 20, 21]. Our interviews revealed a novel finding that discussion of weight during cancer treatment often occurs in the setting of recurrence risk, particularly among participants with breast cancer. Conversations around weight in the context of cancer recurrence should be evidence‐based; while obesity and weight are known risk factors for breast cancer recurrence and mortality, [1, 43, 44] sparse literature indicates that weight loss actually reduces the risk of cancer recurrence, and recent ASCO guidelines recognize that there is insufficient evidence to recommend weight loss or weight gain prevention interventions during cancer treatment [45]. Indeed, one study even suggested that weight loss may *increase* the risk of death after a breast cancer diagnosis [46]. Asking patients to lose weight (something that most have likely tried at many points before [47, 48]) during a time when they are already coping with a cancer diagnosis may also cause additional harm or distress to the patient. Further, given that our interviews indicated that patients may view weight loss as a silver lining of cancer, clinicians may consider educating patients and recommending against weight loss. Additional research on this topic is sorely needed to both provide evidence‐based information on the relationship between post‐diagnosis (deliberate) weight loss and recurrence/mortality risk, as well as how to present this information in a way that does not increase shame or fear among patients.

Our findings are in general agreement with a small body of literature that explores patient experiences of weight stigma across the cancer continuum. A recent systematic scoping review from our group [24] revealed five qualitative studies on this topic, two focused on screening, one on treatment, and two on survivorship. Across these studies, discriminatory behaviors reported included patients not being offered non‐curative therapies because of their body weight, treatment delays, poor treatment by clinicians, and lack of appropriate medical equipment to accommodate larger bodies [34, 35, 36, 49, 50]. These studies also reported patient experiences of weight‐based stereotyping by medical providers and internalization of weight bias that led to shame and healthcare avoidance. New themes emerging from our present study that add to our understanding of this issue include the discussion of weight only as it relates to cancer recurrence (discussed above) and the impact of internalized bias on the patient's ability to recognize experiences of stigma. A growing body of literature exists on weight bias internalization and its impacts [51, 52, 53] but little in the context of cancer patients and survivors. Thus, there is a growing need to understand and address weight stigma perpetrated across the cancer continuum and how it impacts cancer care and outcomes.

We theorize that weight stigma may be more likely to occur at certain points across the cancer continuum and that this may impact cancer‐related healthcare in different ways, as indicated by our main findings. For example, in the primary care setting, the focus is on diagnosing and managing a wide range of conditions, including both obesity and cancer screening. Given prevalent attitudes about obesity and its costs and consequences [54, 55, 56] as well as obesity measurement and counseling being part of measures such as the HRSA Health Center Program Uniform Data System, weight management is likely a high priority for many primary care providers. In our interviews, the primary care setting was often mentioned as a space where participants experienced discussions of weight, and also weight stigma. Cancer screening for women in larger bodies may also be perceived by providers as technically difficult [57] and women in larger bodies may be less likely to be offered cancer screening [2, 26, 28]. In the clinical setting where patient‐provider interaction times are limited, it is possible that cancer screening may be de‐prioritized in favor of discussions of weight. In contrast, after a cancer diagnosis, the focus is on life‐saving care, with perhaps little to no opportunity to discuss weight management, and/or for physicians' personal biases regarding obesity to impact care. This is supported by our findings that many women perceived no impact of their weight on decision‐making during cancer treatment. Unfortunately, while we know that approximately two‐thirds of patients in larger bodies report experiencing weight stigma from doctors [21], and a majority of medical providers and trainees report anti‐fat attitudes [58, 59, 60, 61], little research has been conducted to understand the prevalence of anti‐fat attitudes among cancer treatment providers. One survey study among surgical oncologists revealed that one in three would change the timing/sequence of when resection would be offered based on body size, and that many perceived that having a larger body was related to a wide array of intra‐ and postoperative adverse outcomes [62] despite mixed evidence on the topic [63, 64, 65, 66]. Research is sorely needed to understand when and how often weight stigma is occurring across the cancer continuum, the prevalence of anti‐fat biases among oncologists, and the impact of biases on the delivery of cancer care.

This study has several limitations that warrant consideration. First, we recruited participants based on BMI taken from height and weight reported on driver's licenses. While it is probable that this resulted in some misclassification of those with and without obesity, we confirmed participants' height and weight at the time of diagnosis during the interview. Additionally, we acknowledge that BMI has well‐known limitations as a measure of obesity and is not currently recommended for use as a stand‐alone metric in clinical practice [67]. Nevertheless, we chose to use this measure because clinical decision making remains guided by BMI. Next, while qualitative data collection provides an opportunity for more in‐depth and detailed data collection to occur, eliciting responses to emotionally charged questions about a topic that is stigmatized can sometimes pose concerns related to social desirability bias or acquiescence in responses. Our interviews were conducted over the phone rather than in person, which may have mitigated some of these concerns. Additionally, since we were recruiting through the cancer registry, diagnoses were several years prior to the interview, which could have resulted in some mis‐recollection. We restricted our inclusion criteria to a short time period (diagnosis year 2017–2019) to reduce the possibility of recall bias differential by time since diagnosis. Finally, because of the qualitative nature of this work, we were not able to assess the prevalence of stigmatizing experiences across the cancer continuum; qualitative studies using measures such as the Weight Stigma in Healthcare Inventory [68] are sorely needed to understand the pervasiveness of this issue.

In this study to identify experiences of weight stigma among breast and cervical cancer survivors in larger bodies, we found that while many women did not report an impact of their weight on cancer treatment, those who did experience stigma experienced a wide range of stigmatizing experiences ranging from environmental stigma to interpersonal. We also identified many misconceptions around the causes and consequences of obesity that were held by both the participants of this study and their providers. Several gaps and areas for future research are needed to comprehensively address the impacts of weight stigma on cancer patients and their cancer outcomes, including the need for quantitative research to assess the prevalence of experienced weight bias (including internalized bias) by patients across the cancer continuum; the need to explicitly compare experiences across the continuum; and the prevalence of anti‐fat attitudes held by cancer treatment providers, and whether/how such attitudes shape treatments received.

## Author Contributions

Project conceptualization done by K.M.R., M.E.C., I.M.L., and S.H.N. Methodology done by J.L.S. and S.H.N. Data curation and investigation completed by J.L.S. Formal analysis completed by J.L.S. and S.H.N. Supervision and funding acquisition done by S.H.N. Project administration conducted by J.L.S., M.M.W., and S.H.N. Original manuscript written by J.L.S. and S.H.N. All authors reviewed and edited the manuscript.

## Conflicts of Interest

The authors declare no conflicts of interest.

## Supporting information


Table S1.

## Data Availability

Interview data generated and analyzed during this project are not publicly available but are available from the corresponding author upon reasonable request.
