# Supplementary material for: Patient Perspectives on Impact of Weight and Weight Stigma on Breast and Cervical Cancer Treatment: A Qualitative Study
Source: Cancer Med. 2025 Mar 20;14(6):e70823. doi: 10.1002/cam4.70823 (PMC11924286; doi:10.1002/cam4.70823)
Supplement: Supplementary file 1 — Table S1. [file CAM4-14-e70823-s001.docx]

**Supplementary Table 1: Interview questions and their rationale for inclusion**

| Interview question | Question rationale |
| --- | --- |
| How long has it been since your cancer diagnosis? Can you tell me a little bit about how you’ve been since your cancer diagnosis and treatment? | Icebreaker question(s). Designed to ease participants into the interview and to establish interviewer-interviewee relationship. |
| To start, I’d like to talk more generally about experiences you may or may not have had related to your weight. Has there ever been a time when someone has treated you differently because of your weight? Can you tell me about that experience? *How did that experience make you feel?* | To answer the research question, “*How have breast and cervical cancer survivors with obesity experienced weight stigma in their daily lives?”* |
| Can you recall a time when someone in the healthcare setting - so a doctor, a medical assistant, or similar - treated you differently because of your weight? Can you tell me about that experience?  *If needed, probe with:* Where did this experience take place? (e.g., primary care, cancer screening, cancer diagnosis, cancer treatment, other specialty care) What did this experience feel like for you?  How did you respond to this experience? What did you do? | To answer the research question*, “How have breast and cervical cancer survivors with obesity experienced weight stigma in the healthcare setting, where (e.g., primary care, specialty care, etc.) these experiences took place, and what they were like for the participant?”*  Question included to ascertain whether participants understood the concept of weight stigma and could report prior experiences. |
| Can you recall a time when someone in the healthcare setting - so a doctor, a medical assistant, or similar – provided you with really good care that was sensitive to your weight? Can you tell me about that experience?  *If needed, probe with:* Where did this experience take place? (e.g., primary care, cancer screening, cancer diagnosis, cancer treatment, other specialty care) What did this experience feel like for you? How did you respond to this experience? What did you do? | To answer the research question, *“How have breast and cervical cancer survivors with obesity had body positive experiences, or positive experiences within the healthcare setting? Where (e.g., primary care, specialty care, etc.) and what were these experiences like?”*  Question included to ascertain whether participants understood the concept of weight stigma, and could report prior experiences. |
| Now I’d like to shift and talk specifically about the process of cancer diagnosis and treatment, and what that was like for you. Thinking back to when you received your cancer diagnosis and treatment, what challenges did you experience? *Tell me more about that.* | To answer the research question, *“What barriers to receiving a cancer diagnosis and/or treatment did the individual experience? How did weight stigma impact these barriers?”* |
| Did you ever feel like your weight was a barrier to getting your cancer diagnosed or treated? *What made you feel this way?*  *Were you ever unable to have any medical tests or procedures as part of your diagnosis, because of your weight?*  *How did your weight impact your ability to get diagnostic tests or procedures?* | To answer the research question, *“What barriers to receiving a cancer diagnosis and/or treatment did the individual experience? How did weight stigma impact these barriers?”* |
| Do you recall any times during your diagnosis or treatment for cancer that a member of your care team treated you differently because of your weight? *Can you tell me more about this experience? What did that feel like for you?* | To answer the research question*, “How have breast and cervical cancer survivors with obesity experienced weight stigma in the healthcare setting, where (e.g., primary care, specialty care, etc.) these experiences took place, and what they were like for the participant?”* |
| Was your weight ever mentioned during any decision-making processes regarding your cancer treatment? *Do you know if there were ever any treatments that you were unable to have because of your weight?* | To answer the research question, *“How did the individual’s weight affect their cancer diagnosis or treatment, or their interactions with healthcare providers during the diagnosis or treatment process?”* |
| Did your own feelings about your weight ever impact your cancer care? *Tell me more about that.* | This question sought to examine whether an individual’s perception of obesity or internalized weight bias may have impacted their cancer care. |
| Is there anything you would like to add that we haven’t talked about? | A final opportunity to share any insight that was not captured during the interview. |
| Are you willing to share with me the following information that will help us during data analysis? (Collect information on age, confirm height and weight at time of diagnosis, gender, and cancer site diagnosed) | This question provided participant characteristics that were needed for analysis. We de-identified our interviews and as such, could not link them back to the original data pull/ eligibility information for each participant. |
